# Assessment model blending formative and summative assessments using the SOLO taxonomy

**DOI:** 10.1111/eje.12787

**Published:** 2022-02-27

**Authors:** Gunnel Svensäter, Madeleine Rohlin

**Affiliations:** ^1^ Faculty of Odontology Malmö University Malmö Sweden

**Keywords:** assessment, dental education, feedback, learning, SOLO taxonomy, understanding

## Abstract

**Introduction:**

Formative assessment with emphasis on feedback has been linked to developmental purposes of assessment, whilst summative assessment is assumed to focus on judgemental and quality assurance purposes. This dichotomy is questioned but designs to blend formative and summative assessments in constructive ways are rare in health care education.

**Materials and Methods:**

We have designed an assessment model blending formative and summative assessments. In the formative assessment at the end of a course, students’ responses to real‐life scenarios with questions demanding responses at the relational level of understanding were assessed at three levels of understanding (incorrect, descriptive and relational) modified after the SOLO taxonomy. Students were presented with individual feedback for each response. At the summative assessment of a subsequent course, the students’ new responses were assessed underpinning a final judgement of students’ performance. The assessment model was justified across three student cohorts.

**Results:**

Both formative and summative assessment events of the model provided information about the levels of understanding, unique to each student. A comparison of results from the assessments demonstrated that most responses developed to a higher level of understanding. With the summative assessment, it was possible to make judgements about whether or not individual students passed the pre‐set standards.

**Conclusions:**

We argue that the current assessment model presents real interdependence between formative and summative assessments and can provide information that meets the needs of students as learners, education institutes and health care organisations. The SOLO taxonomy can be used to emphasise the importance of developing and assessing cognitive complexity.

## 1 INTRODUCTION

### 1.1 Blended formative and summative assessments

In this study, we apply two educational concepts: (i) blended formative and summative assessments as an assessment model and (ii) the Structure of the Observed Learning Outcomes (SOLO) taxonomy as a method to assess students’ level of understanding. The first section of the Introduction addresses a short background to blended formative and summative assessments, and the second section below introduces the SOLO taxonomy. Educators and students most often associate assessments with judgements about whether or not the students have met the requirements for passing a course. Besides the judgemental purpose, that is, a summative assessment providing information that enable judgements of learning in relation to intended learning outcomes, there are other purposes of assessment.^1^ These are developmental purposes, that is, the formative assessment to provide feedback to students for learning and the evaluating purpose, that is, an evaluation of courses or programmes providing information that enable quality enhancement. Feedback is a key element of formative assessment as it supports cognitive and professional development. Instead of connecting assessment formats and allowing them to work in harmony, educational research has unintentionally created a harmful and false dichotomy between formative and summative assessments during the last decades.^2^ More recently, however, educational research has focussed on the complementary characteristics and interdependence between formative and summative assessments. In a blended form of assessments, formative assessment is a tool to improve students’ summative performance, and formative assessment is in this way a real precursor to summative assessment. Biggs^1^ suggests that educators should synthesise the positive effects of assessments and create a situation where formative and summative assessments support each other as a tool to enhance students’ process of understanding and thinking. Despite numerous arguments and evidence of the benefits of reconnecting and promoting a blended model for formative and summative assessments, there are few studies within health care education illustrating practical implementation of this approach.^3^, ^4^, ^5^

### 1.2 The SOLO taxonomy

Assessment, whether formative or summative, should preferably be designed in order to find out how students understand and interrelate topics. The SOLO taxonomy, short for “Structure of the Observed Learning Outcome,”^1^ distinguishes between five levels indicated by a hierarchy of verbs according to cognitive complexity (Table 1). The word structure signifies that it is the knowledge structure that is being assessed, regardless of subject matter or domain. Assessment practice may foster two main changes in the outcomes of student learning: *quantitative*, as the amount of facts, and *qualitative*, as structures when knowledge becomes integrated and students demonstrate a level of understanding.^1^ As understanding develops gradually, it is expected to become more structured from the first to the higher grades of an educational programme. The SOLO taxonomy has been used in different disciplines at different levels in higher education.^6^ To the best of our knowledge, the concept of the SOLO taxonomy has not been applied in dental education to assess student responses concerning the development of level of understanding from one course to a subsequent course.

**TABLE 1:** Two phases and five levels of understanding with verbs describing the levels adapted after the SOLO taxonomy^1^

Level of understanding				
Prestructural	Unistructural	Multistructural	Relational	Extended abstract
misses points uses irrelevant information	deals with one relevant aspect memorise name define	combine classify list enumerate	analyse compare contrast relate explain causes	theorise generalise reflect hypothesise
	*Quantitative phase* Students capable of dealing with one or several issues, but these are considered independently to one another		*Qualitative phase* A shift when students demonstrate how various aspects interact	

### 1.3 Educational context

The actual dental programme is constructed according to the principle of a “Spiral curriculum”^7^ that provides learning outcomes and assessment tasks with an increasing structural complexity from the first to the fifth year of the programme that includes ten full semester courses, each devoted to an integrated theme. Students’ learning is assessed in summative assessments at the end of each course resulting in a grade of pass or fail. The summative assessment includes varied assessment formats with a multidisciplinary approach mimicking professional situations rather than separate tests in different subjects. The summative assessments include mandatory student evaluations of each course with written and oral questions on how students perceive their competence in relation to each learning outcome as well as in more general terms. A written summary of the evaluation is forwarded to the undergraduate committee providing information for continuous quality improvement (CQI).

For each year of the programme, an assessment group with a chairperson is appointed by the undergraduate committee and tasked with designing, agreeing upon and then monitoring the end‐of‐course assessments in alignment with the learning outcomes. Each group consists of 6–8 members representing different knowledge fields. A newly appointed chairperson proposed that there was a rationale in line with CQI to improve the written part of the assessment to clarify students’ levels of understanding. The assessment group agreed with the chairperson that previous assessments were based mainly on factual recall questions. After an introduction of the SOLO taxonomy, it was agreed amongst the assessment group that as SOLO presents levels of understanding, this taxonomy may be fit for the purpose. It was also agreed that it was motivated to provide feedback to the students about their level of understanding and then judge the outcomes in a follow‐up assessment.

### 1.4 Aims of the study

An assessment model was designed to assess students’ responses regarding their levels of understanding using a framework based on the SOLO taxonomy. To promote students’ learning as well as certifying students’ performance, the model described sought to balance the perspectives between developmental and judgemental purposes by blending formative and summative assessments.

We aimed to justify the assessment model by exploring the following regarding assessment:

- the developmental purpose by assessing the development of students’ level of understanding. Development should manifest itself as an increase in the level of understanding from baseline to follow‐up,
- the judgemental purpose by assessing whether students had improved their level of understanding and met the requirements for pass/fail,
- the evaluating purpose by finding out about the courses: what works well and what does not work.

## 2 METHODS

### 2.1 Participants

We conducted the study with last‐year students, who attended two consecutive courses, hereafter referred to as Course I and Course II for simplicity. The assessment model was tested in three student cohorts that graduated in 2018, 2019 and 2020.

### 2.2 The present assessment task

**FIGURE 1:**
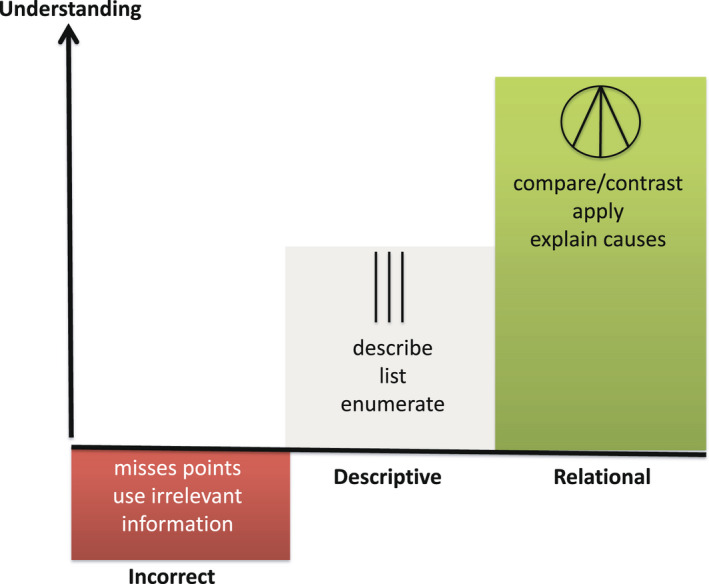
Three categories describing levels of understanding with a hierarchy of verbs modified after the SOLO taxonomy^1^ used to assess students’ responses

The assessment of the last year contained varied formats: a clinical examination with internal and external examiners, oral and written assessments and a degree project. Accordingly, the present assessment task was one part of the complete assessment and was presented in the two consecutive courses and designed for written assessments. The written assessment consisted of 5 key feature scenarios designed to mimic students’ future practice. Each scenario contained four open‐ended questions that should illustrate the holistic nature and be of equal importance. Two to three members of the assessment group were responsible for designing one scenario with questions and model answers aligned with selected learning outcomes. To ascertain students’ levels of understanding, their responses were to be categorised in three levels of understanding modified after the SOLO (Figure 1). The formulation of the questions should therefore be carefully designed in order to stimulate students to elaborate and respond at the relational level.

**FIGURE 2:**
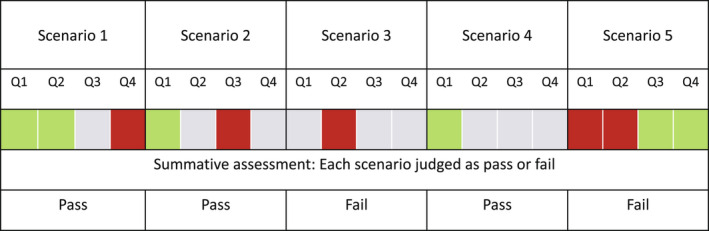
Upper part of the template presents an example of the formative assessment with levels of understanding as presented in Figure 1 for each response of one student. Each student received a template unique to the student as feedback. The lower part of the figure presents how the summative assessment of each scenario was made for pass or fail based on levels of understanding

To make sure that there was a solid and stable categorisation of students’ responses, the assessment group went through a number of control measures. First, students’ responses, which are anonymous and coded, were read and categorised in parallel by the persons responsible for a scenario using the model answers and matched criteria. Second, the categorisations were compared and discussed amongst two or three people to reach consensus and the motivation for the categorisation was noted. The results were collected by the chairperson of the assessment group and an administrator, who produced a written overview of the complete assessment. After the formative assessment of course I, regarded as the baseline, each student received a template with feedback with categories as presented for each response (Figure 2) to support their development of level of understanding at the summative assessment of course II regarded as the follow‐up of development. Each student also received a similar template with feedback for each response after the summative assessment. This meant that students could compare the levels of understanding received at both assessments and judge whether and how they developed or not.

### 2.3 Educational context for the assessment model

The formative assessment at the end of Course I had a developmental purpose, and the summative assessment at the end of Course II had a judgemental purpose. At the beginning of Course I, the students were informed about the written assessment and explicitly told that they were to be given feedback to their responses of each question. The feedback would consist of one of three categories representing level of understanding according to Figure 1 that were presented to them and discussed. They were informed that the assessment at the end of Course II would consist of the same scenarios and questions together with a few additional scenarios. During a 2‐month period after the assessment of Course I, students were free to use whatever resources they chose to develop their understanding. At the summative assessment at the end of Course II, the students were expected to present responses at higher levels of understanding in order to pass the course.

The summative assessments were criterion‐referenced, that is, pre‐set criteria for pass or fail were aligned with a sample of learning outcomes. As the students were to graduate immediately after the assessment of Course II, it was considered relevant to include selected learning outcomes of the Swedish Degree of Master of Science in Dental Surgery according to the National Higher Education Ordinance.^8^

### 2.4 Developmental purpose of the formative assessment, that is, assessment for student learning

In the formative assessment, students’ responses in Course I were used to give feedback to the students. Analysis of the developmental purpose of the formative assessment consisted of the categorisation of each student's responses into the predetermined three categories of levels of understanding presented in Figure 1. Changes in categories to a higher level of understanding from the formative (baseline) to the summative assessment (follow‐up regarded as outcome) were rated as development.

### 2.5 Judgemental purpose of the summative assessment, that is, assessment of student learning

At the summative assessment, final judgement (pass/fail) was based on pre‐set criteria for students’ total performance of the scenarios. Pass was given when there was one response at the relational level and at the same time a maximum of one response at the incorrect level (Figure 2). Scenarios with responses only at the descriptive level or with two responses at the incorrect level were assessed as fail (Figure 2). Students’ total performance was judged as pass or fail. All five scenarios were designed to mimic authentic problems in the students’ future professional life and to be important. Therefore, a scenario assessed as failed could not be compensated by another scenario with responses at the relational level. To achieve pass, all the scenarios should be assessed as pass whilst the total judgement was a fail when one of five scenarios was assessed as fail.

### 2.6 Evaluating purpose of assessment for quality enhancement

Information for the evaluating purpose was retrieved by the assessment group and by the students. The assessment group reviewed students’ responses in order to identify domains that students appeared to understand at a higher vs. lower level. In the written course evaluation, the students were asked to state their level of agreement with a series of questions that were linked to the learning outcomes and the assessments. The response categories—not at all, to a low degree, somewhat, to some degree, to a high degree—were consistent with standardised student evaluation tools used in the university and therefore familiar to the students. The students and the chairperson of the assessment group then discussed strengths, weaknesses and opportunities for enhancement of the courses.

## 3 RESULTS

### 3.1 Developmental purpose of assessment for learning

**FIGURE 3:**
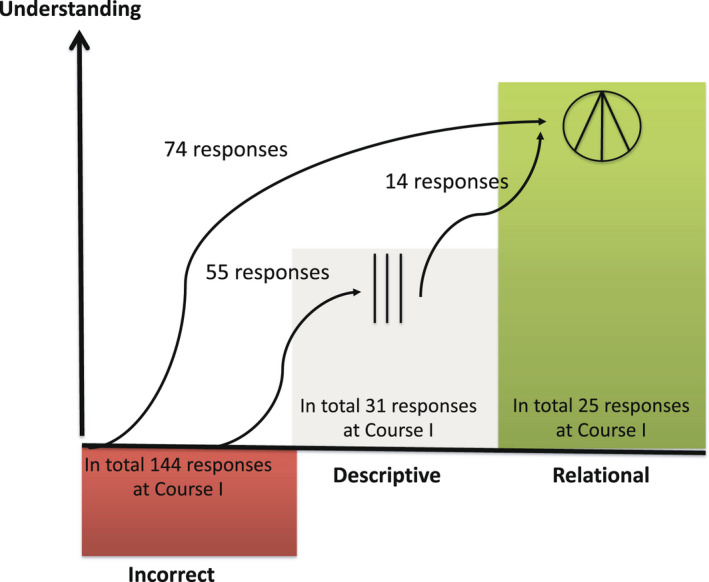
Number of responses that were assessed to have developed to a higher level of understanding from the formative assessment (Course I) to the summative assessment (Course II) for one student cohort

Assessment of students’ responses to the same questions at the end of course I and II, using the given SOLO‐categories, made it possible to evaluate the development of students’ understanding over time. The number of responses that developed to a higher level of understanding for the student cohort that graduated 2020 is presented in Figure 3. Seventy‐four of 142 responses developed from incorrect to the highest level of understanding (relational) and 55 responses to the descriptive level. In total, 81% of students’ responses at Course I was assessed as having reached a higher level of understanding at Course II.

A similar trend in the development of understanding was seen for the two other student cohorts. As presented in Table 2 for three student cohorts, the level of understanding was assessed to develop on average in 75% of the responses: 43% (range 37–51) from incorrect to relational, 17% (range 9–28) from incorrect to descriptive level and 15% (range 7–24) from descriptive to relational level of understanding. Altogether, 15% (range 7–19) of the responses did not demonstrate a development in the level of understanding (Table 2).

**TABLE 2:**
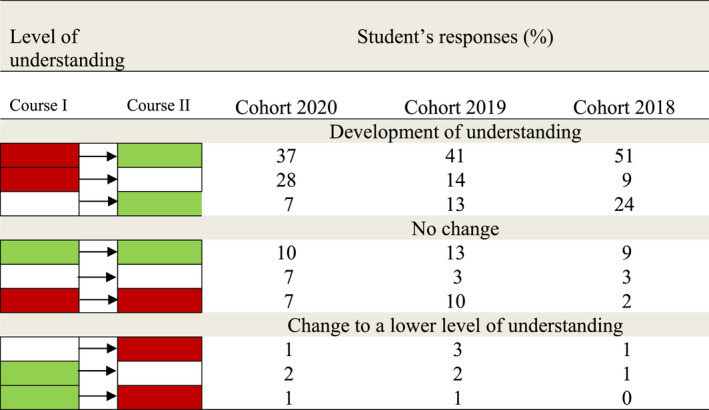
Comparison of students’ responses given at formative assessment (Course I) and summative assessment (Course II) as categorised in levels of understanding presented as percentage (%) of total number of responses of three student cohorts (graduation year 2020, 2019, 2018). Levels of understanding: relational []; descriptive []; incorrect []

### 3.2 Judgemental purpose of assessment of learning

**FIGURE 4:**
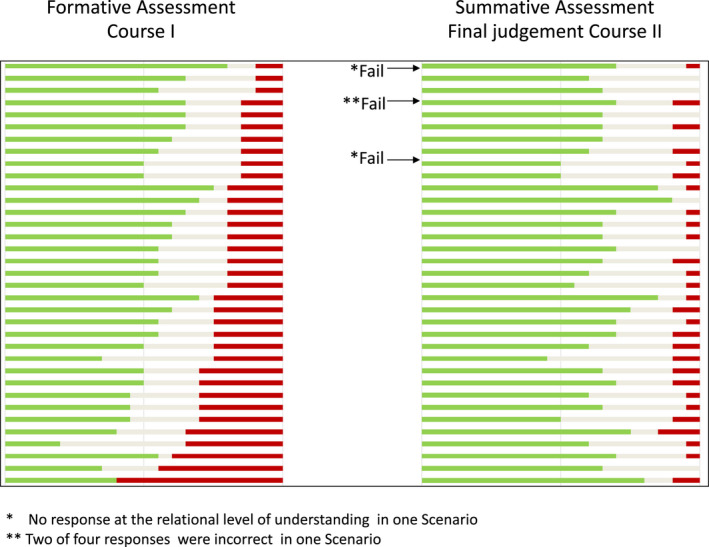
Formative assessment and summative assessment for individual students (each student is represented by a horizontal line) of one student cohort demonstrating responses at relational [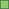], descriptive [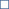], or incorrect [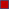] levels of understanding. Many incorrect responses at the bottom of the formative assessment developed to the relational level of understanding at the summative assessment. Many responses at the descriptive level remained at the same level

Based on the summative assessment, the judgemental purpose of assessment could be justified. The results of the summative assessments of one student cohort are presented in Figure 4. Overall, the majority of responses were at the relational level with variation amongst individual students. Some responses were at the descriptive level, and a few responses were at the incorrect levels. In this cohort, the total performance of three students was judged as a fail according to the pre‐set criteria for pass/fail (Figure 2). Two students presented no response at the relational level in one scenario, and one students presented two responses at the incorrect level in one scenario each. As indicated by the responses, not all students were motivated to identify the need for further knowledge for all five scenarios.

The formative and summative assessments presented in Figure 4 illustrate the development of the level of understanding of one cohort and of individual students relative to their colleagues. At the formative assessment, there were large differences amongst students with some students presenting many responses at the relational level, whilst other students presented many responses at the incorrect level. The main change that occurred in responses between the formative and summative assessment was that responses at the incorrect level changed to the relational level. The proportion of descriptive responses was still almost unchanged for the three cohorts.

### 3.3 Evaluating purpose of assessment for quality enhancement of the courses

The assessment group identified that many responses on risk assessment and diagnostic thinking were at the incorrect and descriptive levels at the formative assessment and that the development of level of understanding as assessed in the summative assessment was less than outstanding. Students’ achievements and their own evaluation of their competence in these two fields were not correlated. For other learning outcomes of the courses, there was a high agreement between students’ evaluation and the reality, that is, students’ performance. Students’ written answers to two questions focussing on the assessments are presented in Table 3. About 86% of the students perceived the formative assessment as supporting their learning and 76% perceived the summative assessment as judging their learning and understanding.

**TABLE 3:** Students’ perception of assessments in Course I and Course II. Results are presented as mean in percentage of responses of all students of three cohorts

Students’ perception of assessment				
To what degree did the assessment in Course I support your learning for the following assessment in Course II?				
Not at all 0%	To a low degree 11%	Somewhat 3%	To some degree 48%	To a high degree 38%
To what degree did the assessment in Course II judge your learning and understanding?				
Not at all 0%	To a low degree 5%	Somewhat 19%	To some degree 46%	To a high degree 30%

When expressing themselves in the oral discussion with the chairperson of the assessment group, the students were outspoken and enthusiastic about their opportunities to influence the programme, including the assessment model. They stated that they considered the feedback to be honest and relevant. Although the majority perceived the levels of understanding to be relevant for the judgement pass/fail, they expressed that the descriptive level may be sufficient. All students stated that they considered the individual feedback after the summative assessment to be valuable as they were eager to find out about the development of their understanding.

## 4 DISCUSSION

The purpose of the current study was to describe and justify an assessment model by addressing the research question “Does blended formative and summative assessments using SOLO taxonomy work?” Since description and justification studies should be based on previous conceptual frameworks,^9^ we build our model on the results of previous educational research regarding assessment, feedback and the SOLO taxonomy. Students’ responses to open‐ended questions were assessed in two subsequent courses using a modified SOLO taxonomy with three levels of understanding (incorrect, descriptive or relational). The feedback of the formative assessment worked as most responses developed from the incorrect or descriptive to the relational level of understanding. The summative assessment made it possible to make judgements about whether or not individual students met the pre‐set standards. These outcomes are based on the implementation of the assessment model of one dental programme. It had been desirable to compare the implementation and the outcomes of the model achieved in other programmes in higher education.

### 4.1 Methodological considerations

The SOLO has been implemented in various disciplines at different levels in higher education for formulating and assessing learning outcomes.^10^, ^11^, ^12^ In health care education, SOLO was implemented in anatomy,^13^ in year two of a medical programme^14^ and in dental education for fourth‐year student's clinical decision making.^15^ As described by Biggs,^1^ the unistructural level is characterised by simple naming and focussing on one issue in a complex case and the multi‐structural level is characterised by a disorganised collection of items and details unrelated to each other. In the current model, these two levels were combined into the “descriptive level” as both indicate only memorisation of one or several issues. When faced with scenarios in health care settings, students’ understanding is supposed to indicate integration and interpretation of information and an understanding of how to apply the information at a relational level. Being assessed on what you have learned becomes a process whereby you have to give explanations about the biological and social aspects of clinical phenomena rather than simply regurgitate facts in an unreflective response.^16^ The fifth level of the SOLO taxonomy,^1^ extended abstract, which is described as relating to existing principles and questioning and going beyond existing principles, was not included as that level is more appropriate for assessing degree projects in the programme.

### 4.2 Developmental purpose of formative assessment

The assessment at the end of Course I corresponds to a formative assessment as it presented information with the purpose of giving feedback to alert students to gaps in their learning and familiarising them with the expectations of the coming summative assessment. The message to the students was that incorrect and descriptive responses were to be developed.

Feedback is a key element of formative assessment as a support for cognitive and professional development.^17^, ^18^ It is worth noting that we as teachers believe that we give feedback on a regularly basis but that the students do not always recognise this.^3^, ^19^ The feedback in the present study comprised visualised levels of understanding (in colours and verbs) to facilitate students in their own revision (elaboration). Thus, the feedback was not directive in a manner that informed the students of what points required correction (verification). Even though the current students had been exposed to multiple sessions with feedback, this was the first time that the feedback indicated that their responses demonstrated an insufficient level of understanding. A proportion of descriptive responses remained on the same level after the feedback signalling that students accepted this level of understanding and were not engaged in making any further developments. These results indicated that it is necessary to elaborate with individual students in a more explicit way whether they reach a sufficient level of understanding or not.

The feedback seemed on the contrary to have an impact on incorrect responses that to a high degree reached a higher level of understanding. This is in contrast to the result of an assessment study in immunology with similar design showing that the formative assessment led to little improvement in the final exam performance.^3^ In our opinion, being exposed on multiple occasions to these types of assessments with SOLO‐levels and feedback may aid students to develop their understanding of knowledge and learning.

### 4.3 Judgemental purpose of summative assessment

The summative assessment at the end of Course II had a judgemental purpose, that is, it was possible to judge whether students individually performed in relation to a sample of learning outcomes. At the same time, it enabled judgement of their development as assessed with the three categories for level of understanding. The approach with identical questions and criteria in line with SOLO‐categories for both assessments facilitated the judgement of whether or not the students’ level of understanding had developed. Thus, the problem with regard to measurement was to estimate for an individual student what fraction of a knowledge domain the student had acquired, or what fraction of a domain of skills the student had mastered based on a sample of test items or tasks.^1^ This standard model of assessment is designed for assessing students’ learning in relation to learning outcomes and part of constructive alignment in an educational programme. It may be tempting to throw grades into the pot and decide that responses at the descriptive level are sufficient and responses at the relational level get an additional grade. Whilst this may be convenient, it is misleading as to the importance of students’ understanding.^1^ Which are the standards at an acceptable level? In the current assessment model, students were expected to present responses at the relational level of understanding being able to integrate topics and use knowledge, which is probably the main aim of most programmes in higher education.

The judgemental purpose was achieved also for one of the learning outcomes as presented in the National Higher Education Ordinance for the Degree of Master of Science in Dental Surgery^8^: “The student shall demonstrate the ability to identify the need for further knowledge and undertake ongoing development of his or her skills.” Although this may be one of the most important learning outcomes for students’ future professional life, we claim that the students’ achievement of this learning outcome is mostly not explicitly assessed.

### 4.4 Evaluating purpose, that is, finding out about the courses/programme

Shepard^20^ highlights the importance of using assessment not only to assess student learning, but also to examine and improve educational practices. The current assessment model revealed issues about the actual educational environment. The proportion of unchanged responses at the descriptive level of understanding disclosed that students were not motivated to develop all their responses to the relational level, which we had hoped that they would do. As the goal of the programme is to trigger engagement in learning activities and to reach some sort of “performance of understanding,”^1^ it is fundamental to consider why the students are not motivated to develop their responses to the relational level and why they are of the opinion that the descriptive level is sufficient. Being able to integrate topics and transfer knowledge to different situations in practice, that is, reaching the relational level of understanding, is probably the main goal of most dental educational programmes. What is “acceptable”?

The assessment model also revealed that a few topics, such as risk assessment and diagnostic thinking, were cumbersome for students of the three cohorts. Judged from the students’ own evaluation, they were unaware about their gap of competence. The credibility of the dental education lies very much in the clinical setting, which has been described as a “culture of certainty.”^21^ On the contrary, diagnostic thinking, and in particular prediction, is linked to uncertainty, which is challenging to learn and teach. Knowledge is more likely to transfer if students have the opportunity to practice with a variety of applications whilst learning.^16^, ^20^ Students’ encounters with a limited variety of patients may, however, restrict transfer of knowledge if the educators do not exploit and support students in recognising different aspects of each encounter, denominated as new ways of seeing.^16^

Discussion of how the current assessment model and the results of this study have informed our future dental programme is beyond the scope of this paper. Yet, it is important to acknowledge that curriculum modification, in particular regarding risk assessment and diagnostic thinking, took place. As we believe that repetitive seminars in the clinical setting underpinning reflection‐in‐action and reflection‐on‐action directly related to students’ own patient encounters support students’ development of understanding, these activities were increased.

### 4.5 Educational implications

The current assessment model takes into account evidence for a paradigm shift in the assessment culture emphasising a blended perspective of formative and summative assessments. The critical component of the model is not using different tools but rather conceptualising assessment as a communication process with feedback to students about their level of understanding. The role of feedback in both the formative and summative assessments fall under the category *longitudinal development* (feed‐forward)^19^ with focus on students’ development of cognitive skills. The summative assessment in this model also includes feedback and aims for a longitudinal development to provide guidance in order to support graduates’ future learning as reflective practitioners.^22^ For students to benefit from this type of assessments with feedback, they must be able to discern an impact on their actions and learning. To be able to do this, students’ ability to reflect and have an understanding of the learning process must be reasonably well developed. If feedback is interpreted only as a justification of the mark awarded, there may be a limited developmental effect. Whatever role the assessments with feedback are intended to have, it should be clear which purposes and rationales are to be achieved and students should be able to recognise the benefits they provide.

Selecting assessment methods involves compromises and assessment characteristics are weighed differently depending not only on the purposes but also the educational context for the assessment.^23^ Regardless of educational context, however, the advantage of SOLO is that student responses can be related to levels of cognitive complexity, which has been observed to correlate with deep and surface learning approaches.^24^ For dental education consisting of a coherent programme of 4–5 years, the SOLO taxonomy constitutes an optimal tool for longitudinally following students’ conceptual change from the first year to graduation. By doing this, we can define the understanding appropriate to the year of a degree programme, as intended by Biggs.^1^ To have a clear picture of the level of understanding that students should be at throughout a programme allows the faculty as an educational community to maintain focus and direction in the creation of learning outcomes, content, assignments and assessments in a holistic way.

When applying an analytical method for assessment of level of understanding, the alignment between situations selected for the assessment and the goodness of fit^1^ with the intended learning outcomes is crucial. In a dental programme, these may present authentic real‐world situations unfamiliar to the students but designed to draw upon their prior knowledge. This is in line with a general approach within higher education stating that: “We have to find out and assess the ways in which students applies understanding to different, preferable unfamiliar, situations…..”.^16^ To achieve this, the formulation of questions with model answers has to be carefully considered. The formulation of questions is critical when students’ responses are expected to demonstrate a level of understanding, that is, being able to integrate topics and use knowledge. Even if we need to be realistic about what most students will be able to achieve for all situations, the assessment tasks, open‐ended question included, should give students the opportunity to respond and assess at a higher level of understanding. If too narrow, the questions will probably not require students to respond at a high level of understanding.

## 5 CONCLUSIONS

We designed an assessment model that presents real interdependence between formative and summative assessments and that can provide information that meets the needs of both students as learners, education institutes and health care organisations. The model was investigated in three student cohorts, and students’ responses to open‐ended questions were assessed using the SOLO taxonomy. The results demonstrated that the developmental purpose of formative assessment was achieved with support from the summative assessment, and at the same time, the judgemental purpose of summative assessment was founded on the formative assessment. The concept of the SOLO taxonomy can be applied to provide a picture of students’ level of understanding. We conclude that alternative assessment strategies may be valuable tools with which to assess learning outcomes and students’ understanding at levels of cognitive complexity.

## CONFLICT OF INTEREST

No conflict of interest exists.

## Data Availability

The data that support the findings of this study are available from the authors upon reasonable request.
